# Physical Growth Dynamics of Children With Juvenile Dermatomyositis

**DOI:** 10.7759/cureus.79861

**Published:** 2025-02-28

**Authors:** Krishna Soni, Harvinder Kaur, Anju Gupta, Surjit Singh, Subhas C Saha, Anil Bhalla

**Affiliations:** 1 Pediatrics, Postgraduate Institute of Medical Education and Research, Chandigarh, IND; 2 Obstetrics and Gynaecology, Postgraduate Institute of Medical Education and Research, Chandigarh, IND

**Keywords:** auxological attainments, india, jdms, physical growth, under-weight

## Abstract

Objective

Growth failure is an often overlooked complication of juvenile dermatomyositis (JDMS) hence, the present study analyses the auxological status of adolescent JDMS patients.

Methods

Sixty serial observations with respect to height, weight, and skinfold thicknesses were made on 35 JDMS children aged 10 to 17 years in the Growth Laboratory/Clinic of the Department following a mixed-longitudinal growth research design. Information with regard to disease duration and disease activity (MMT-8 score) was recorded.

Results

The mean height and weight of JDMS patients showed a regular increase from 10 to 17 years while BMI depicted an undulating pattern. JDMS boys were taller yet weighed lighter and had lesser sub-cutaneous fat deposition than girls. As compared to their normal counterparts, JDMS children were lighter and shorter, however, JDMS girls caught up and became heavier and taller beyond 12 and 14 years, respectively. Only 4 (6.6%) and 5 (8.33%) of our study subjects were short-statured and underweight, respectively. Disease activity had a significant positive correlation with the physical growth of JDMS boys.

Conclusion

JDMS boys depicted compromised growth when contrasted with their unaffected counterparts; this may be due to the influence of disease activity on their physical growth. However, better auxological prognosis recorded for JDMS girls beyond 12-13 years reveals that there must be some protective mechanism favoring girls making them resistant to adverse effects of the disease.

## Introduction

Juvenile dermatomyositis (JDMS) is a systemic inflammatory disorder and vasculopathy that affects children younger than 18 years, having an incidence of two to three children per million per year. It is more common in girls than boys [1]. Apart from systemic complications, lipodystrophy, a recognized complication of JDMS, occurs in 7% to 50% of patients [2,3]. Lipodystrophy (significant loss of body fat) may be generalized or partial; generalized lipodystrophy denotes fat loss from the face, trunk, abdomen and all extremities, while partial relates to fat loss from the upper and lower extremities with relative sparing of trunk and abdomen. A slow and progressive loss of subcutaneous and visceral fat, mostly noticeable over the upper parts of the body and face, is often accompanied with calcinosis, hirsutism, acanthosis nigricans, clitoral enlargement, hepatic steatosis, insulin resistance, abnormal glucose tolerance, and hypertriglyceridemia.

JDMS appears to have a significant impact on the physical growth and psychosocial well-being of the patients [4]. These children can have low self-esteem, feelings of isolation and difficulty with social interactions. The longer the duration of untreated disease, the longer the time to achieve remission and consequently the long-term complications. Though with the advent of immunosuppressive treatments, the survival rate of children with JDMS has substantially improved over the years [5], its impact on their growth is visibly compromised. The inflammatory activity of the disease along with the effect of corticosteroid therapy may be the reason for the hindrance of normal growth noticed in these children [6]. However, due to the rarity of this disease, minimal effort has been put to study the growth-related changes across the globe. However, few attempts made to study the pattern of auxological attainments in JDMS children are noteworthy [7,8]. A previous study documenting the pattern of subcutaneous fat [9], as well as a more recent investigation on pubertal attainments [10] of Indian children with JDMS, was conducted at our center.

Owing to the complete absence of auxological information on adolescents, we attempted to study the pattern of physical growth in children with JDMS representing north-western parts of India following a mixed-longitudinal growth research design. This study will contribute to clinical practice by providing an age and gender-specific database for physical growth parameters among JDMS children.

## Materials and methods

A total of 35 children (boys: 16, girls: 19), between 10 to 17 years of age, diagnosed as cases of juvenile dermatomyositis according to criteria given by Bohan and Peter [11] (set of guidelines used to classify dermatomyositis and polymyositis), comprised material for this mixed-longitudinal study. These children were born to parents belonging to mixed socioeconomic strata and were enrolled in the Pediatric Rheumatology Clinic of the Department of Pediatrics, Postgraduate Institute of Medical Education & Research (PGIMER), Chandigarh. Every patient was measured for different anthropometric parameters in the Growth Laboratory/Clinic of the department at the time of enrolment and after six monthly (±1 month) age interval between January 2021 and June 2022 and was evaluated for disease duration, its activity using Manual Muscle Testing (MMT-8) score. MMT-8 is a set of eight designated muscles tested unilaterally (potential score 0-80), generally on the right side. Approval of the Institutional Ethics Committee, PGIMER was also granted to this study. Enrolment of the study subjects was done after obtaining written informed consent from parents and assent from each participant.

The children with dysmorphic facies and physical disability found to be very sick in the beginning were excluded from the study.

Every patient was measured for height, weight, and skinfold thicknesses (triceps, biceps, sub-scapular, mid-axillary, thigh) using standardized anthropometric techniques and instruments [12, 13]. Additionally, the body mass index (BMI, kg/m^2^) was calculated. Throughout the entire study period, the same instruments were used.

Statistical analysis

The distribution of the quantitative variables was tested with the Shapiro-Wilk test / Kolmogorov-Smirnov tests of normality. Our anthropometric data was skewed and the sample size for each age group was small, so data was written in the form of its mean, standard deviation and median and interquartile range. Non-parametric Mann-Whitney U-test was employed to evaluate gender differences for each growth parameter. Inter-population comparison of weight, height and BMI in our study was done with published data on Indian children by the Indian Academy of Pediatrics (IAP, 2015) [14] using one-sample t-test. Spearman correlation coefficient was calculated to see the relationship between different quantitative data. A p-value < 0.05 was considered signiﬁcant. Analysis was conducted using Statistical Package for the Social Sciences (SPSS) version 22.0 (IBM Corp., Armonk, NY, USA).

## Results

**Figure 1:**
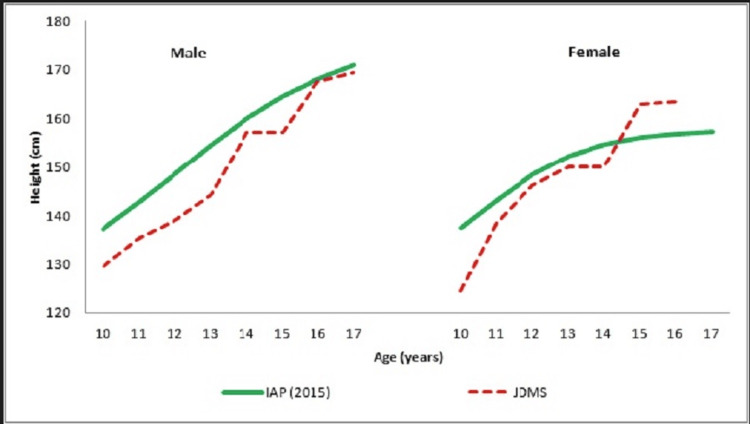
Height (cm) of Male and Female JDMS Patients and Normal Children

**Figure 2:**
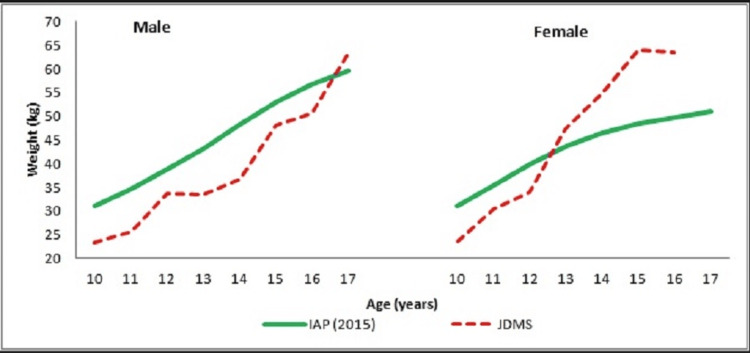
Body Weight (kg) of Male and Female JDMS Patients and Normal Children

The study presents an account of 60 serial observations made on 35 children afflicted with JDMS at half-yearly age intervals. The mean height (cm) and weight (kg) of JDMS patients showed a regular increase from 10 years to 17 years of age (Table 1 & Figures 1-2). Boys with JDMS weighed lighter yet, were taller than their female JDMS counterparts, except between 11 to 13 years where female patients took the lead. Gender differences for body weight and height remained statistically non-significant throughout the study period (Table 1).

**Table 1:** Mean, SD and Gender Differences (Mann-Whitney test) for Height, Weight and BMI of JDMS Boys and Girls *denotes p≤0.05

Age (years)	Height (cm)				Weight (kg)				BMI (kg/m^2^)				Gender Differences (p-value)		
	JDMS Boys		JDMS Girls		JDMS Boys		JDMS Girls		JDMS Boys		JDMS Girls		Height	Weight	BMI
	Mean	SD	Mean	SD	Mean	SD	Mean	SD	Mean	SD	Mean	SD			
10	129.7	15.33	124.5	7.28	23.4	5.02	23.6	5.50	13.38	0.34	15.03	2.16	0.84	0.94	0.94
11	135.3	7.94	138.5	9.18	25.6	6.27	30.5	7.37	13.85	1.67	15.91	3.45	0.56	0.21	0.30
12	139	9.47	146.1	7.21	33.8	0.28	34.0	1.25	17.62	2.54	16.00	1.04	0.24	0.56	0.24
13	144.2	12.94	150.0	0.64	33.6	3.39	47.4	8.45	16.49	4.57	21.06	3.83	1.0	0.08	0.56
14	157.2	6.71	150.0	7.82	36.6	5.58	54.8	15.90	14.76	0.99	23.90	4.63	0.24	0.24	0.05*
15	157.2	8.85	162.8	4.38	48.2	15.22	63.9	10.67	19.26	4.49	24.05	2.75	0.56	0.24	0.24
16	167.4	10.16	163.3	4.38	50.6	15.81	63.4	10.21	17.69	3.21	28.70	9.61	0.69	0.24	0.12
17	169.2	8.13	-	-	63.4	1.87	-	-	21.83	4.36	-	-	-	-	-

JDMS boys representing the present study were shorter compared to their IAP counterparts [14] throughout the ages considered, however, the magnitude of inter-group differences remained non-significant. At the same time, girls with JDMS being shorter initially (until 14 years) became taller than their un-affected peers at ages 15 and 16 years. Inter-group differences among JDMS and un-affected girls were found to be statistically significant at 10 (p≤0.001, CI: -17.24 to -8.44) and at 13 (p≤0.05, CI: -3.73 to -0.53) years. The study subjects were classified as normal (height for age ≥ 3rd centile) and short-statured (height for age < 3rd centile) according to IAP 2015 growth references. Only 4 (6.6%) of our study subjects were observed to be short-statured, out of which three were boys and only one was a girl. As per Mid-Parental Height (MPH), which was available for 13 patients, 4 (31%) were stunted.

In our study, it was found that only 5 (8.33%) JDMS children were underweight (weight for age < 3rd centile of IAP 2015 reference), and interestingly, all of them were males. The mean weight attainment among JDMS boys was compromised when compared to their IAP counterparts [14], except at 17 years of age. However, the magnitude of inter-group differences as determined by the one-sample t-test was significant only at 12 years (p≤0.05, CI: -7.74 to -2.65). While, girls with JDMS, weighed lighter than their un-affected peers only until 12 years whereafter, they became heavier. Inter-group differences were significant at 10 (p≤0.000, CI: -10.71 to -4.06) and 12 years (p≤0.05, CI: -8.87 to -2.66).

**Figure 3:**
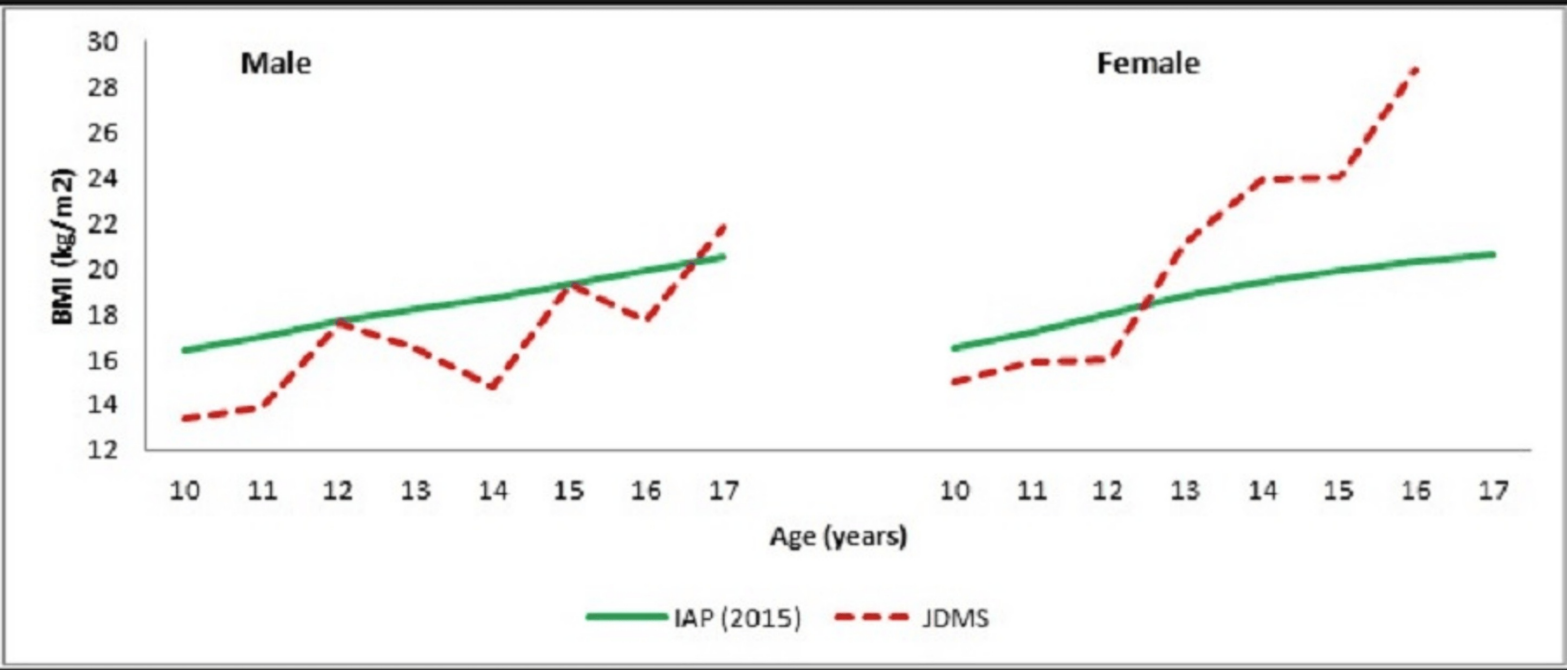
Body Mass Index (kg/m2) of Male and Female JDMS Patients and Normal Children

A continuous increase was noted for mean BMI among female JDMS patients (Figure 3). In contrast, BMI among male patients depicted an undulating growth trend. Though girls with JDMS possessed higher BMI than their male counterparts throughout the study period yet, the magnitude of gender differences reached statistical significance only at 14 years (p≤0.05) (Table 1). As compared to their IAP counterparts, boys with JDMS depicted a compromised auxological status while JDMS girls had higher BMI beyond 13 years. The magnitude of inter-group differences was significant only at 10 years of age. Patients with BMI <3rd centile were classified as thin, 3rd to 23rd were classified as normal, 23rd to 27th were classified as overweight, and >27th centile were classified as obese as per IAP (2015) reference. Accordingly, 55% (n=33) of the study subjects had normal BMI, 28.33% (n=17) were overweight and 10% (n=6) were obese. Only 6.67% (n=4) were noted to be in the thinness category, out of which three subjects were boys.

**Figure 4:**
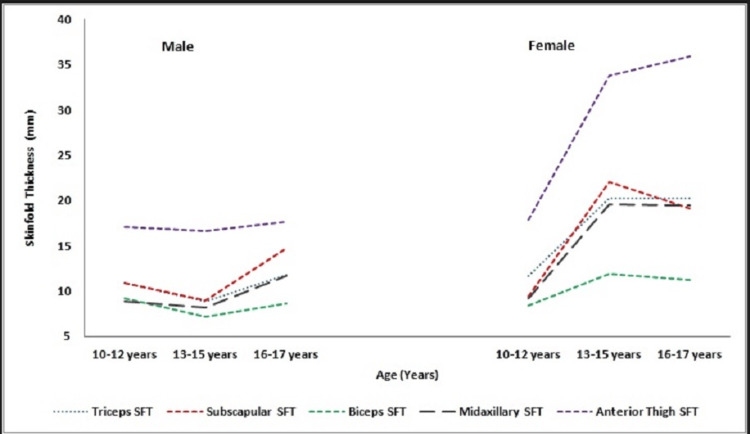
Skinfold Thicknesses (mm) of Male and Female JDMS Patients

The mean and SD of different truncal (sub-scapular and mid-axillary) and appendicular (triceps, biceps, anterior thigh) skinfold thicknesses measured among boys and girls with JDMS are shown in Table 2. As the inter-age differences in the present study obtained by applying the Mann-Whitney test were found to be statistically non-significant, the ages were pooled for various skinfold thicknesses into early adolescence (10-12 years), middle adolescence (13-15 years) and late adolescence (16-17 years) to arrive at better auxological inferences. The greatest deposition of sub-cutaneous fat among our study children was noted at the anterior thigh region while the lowest value was recorded for biceps skinfold thickness (Figure 4). Girls with JDMS were fatter for all skinfolds than their male counterparts with highly significant gender differences recorded between 13-15 years (Table 3).

**Table 2:** Mean and SD of Different Skinfold Thicknesses (SFT) among Boys and Girls with JDMS

Skinfold thickness (mm)	JDMS Boys						JDMS girls					
	10-12 years		13-15 years		16-17 years		10-12 years		13-15 years		16-17 years	
	Mean	SD	Mean	SD	Mean	SD	Mean	SD	Mean	SD	Mean	SD
Triceps SFT	10.87	6.28	8.90	4.05	11.81	4.82	11.67	3.61	20.22	6.32	20.20	3.11
Subscapular SFT	10.85	8.49	9.01	5.63	14.77	8.51	9.47	3.45	22.10	9.31	19.10	1.27
Biceps SFT	9.20	4.37	7.12	3.51	8.62	2.81	8.45	2.67	11.88	2.33	11.20	2.54
Midaxillary SFT	8.90	6.92	8.22	5.53	11.68	6.49	9.23	3.63	19.54	6.97	19.50	3.53
Anterior thigh SFT	17.07	10.81	16.68	9.37	17.70	4.70	17.84	7.04	33.82	9.85	35.90	5.51

**Table 3:** Gender Differences for Skinfold Thicknesses among Boys and Girls with JDMS ** denotes p≤0.01; *** denotes p≤0.001

	Mann-Whitney Test (p-value)				
Age (years)	Triceps SFT	Subscapular SFT	Biceps SFT	Mid-axillary SFT	Anterior thigh SFT
10-12	0.168	0.603	0.982	0.331	0.355
13-15	0.002**	0.002***	0.015**	0.003**	0.008**
16-17	0.085	0.197	0.197	0.197	0.300

Table 4 depicts mean growth velocities calculated for different anthropometric parameters measured at six monthly age intervals among JDMS children enrolled in our study. Female JDMS children possessed higher growth velocity for all the studied parameters except height than their male peers. Statistical analysis, however, depicted non-significant gender differences.

**Table 4:** Mean, SD and Gender Differences (Mann-Whitney Test) for Height Growth Velocity, Weight Growth Velocity, BMI Growth Velocity and Skinfold Thicknesses Growth Velocity in JDMS Boys and Girls

	JDMS Boys			JDMS Girls			Gender Differences (p-value)
	Mean	SD	Median	Mean	SD	Median	
Height velocity (cm/6 months)	1.17	1.83	0.70	0.88	0.85	0.85	0.734
Weight velocity (kg/6 months)	1.35	2.02	0.80	1.47	3.59	0.30	0.349
BMI velocity (kg/m^2^/6 months)	0.35	0.73	0.15	0.79	1.61	0.10	0.612
Triceps Skinfold Thickness (mm/6 months)	1.17	2.05	0.40	1.38	3.32	0.95	0.955
Sub-scapular Skinfold Thickness (mm/6 months)	0.42	1.29	0.40	0.82	2.57	0.20	1.000
Biceps Skinfold Thickness (mm/6 months)	0.71	1.04	0.60	0.87	3.36	0.30	0.712
Mid-Axillary Skinfold Thickness (mm/6 months)	1.04	1.77	1.00	1.72	3.06	1.50	0.515
Anterior Thigh Skinfold Thickness (mm/6 months)	1.44	1.8	1.00	2.11	4.15	0.00	0.139

Non-parametric Spearman’s correlation coefficient was applied to study the strength and direction of association between selected anthropometric parameters with disease activity (MMT-8 score) and duration of therapy in boys and girls with JDMS (Tables 5, 6). While height and weight had a significant positive correlation (p≤0.05) with duration of therapy, disease activity as assessed in terms of MMT-8 score did not have an association with any of the parameters in female JDMS patients. Among JDMS boys, duration of therapy had a significant positive correlation (p≤0.001) with age at diagnosis while being only significantly correlated to height (p≤0.05) in terms of anthropometric parameters. Contrary to the observations made for female JDMS patients, MMT-8 score was found to have a significant positive correlation with height, weight and BMI among JDMS male patients.

**Table 5:** Correlation between Anthropometric Parameters, Disease Activity and Duration of Therapy in JDMS Boys *denotes p≤0.05; ** denotes p≤0.01; *** denotes p≤0.001

		Height	Weight	BMI	Duration of Therapy	Disease Activity (MMT-8 Score)
Height	Spearman’s Rho	1.0	0.90	0.671	0.43	0.485
	p-value	-	0.000***	0.000***	0.029*	0.014*
Weight	Spearman’s Rho	0.901	1.0	0.901	0.35	0.662
	p-value	0.000***	-	0.000***	0.084	0.000***
BMI	Spearman’s Rho	0.671	0.901	1.0	0.319	0.636
	p-value	0.000***	0.000***	-	0.120	0.001***
Duration of Therapy	Spearman’s Rho	0.43	0.35	0.319	1.0	-0.34
	p-value	0.029*	0.084	0.120	-	0.071
MMT	Spearman’s Rho	0.485	0.662	0.636	-0.34	1.0
	p-value	0.014*	0.000***	0.000***	0.071	-

**Table 6:** Correlation between Anthropometric Parameters, Disease Activity and Duration of Therapy in JDMS Girls *denotes p≤0.05; ** denotes p≤0.01; *** denotes p≤0.001

		Height	Weight	BMI	Duration of Therapy	Disease Activity (MMT-8 Score)
Height	Spearman’s Rho	1.0	0.91	0.74	0.39	0.13
	p-value	-	0.00***	0.00***	0.026*	0.46
Weight	Spearman’s Rho	0.91	1.0	0.93	0.39	0.08
	p-value	0.00***	-	0.00***	0.024*	0.63
BMI	Spearman’s Rho	0.74	0.93	1.0	0.30	0.004
	p-value	0.00***	0.00***	-	0.089	0.98
Duration of Therapy	Spearman’s Rho	0.39	0.399	0.30	1.0	-0.34
	p-value	0.026*	0.024*	0.089	-	0.071
MMT	Spearman’s Rho	0.13	0.08	0.004	-0.34	1.0
	p-value	0.46	0.63	0.98	0.071	-

## Discussion

Juvenile dermatomyositis (JDMS) is a multisystem disease that follows a long course with variable clinical outcomes. One of the less studied and often overlooked complications of JDMS is growth failure, which can be influenced by several factors such as the age at diagnosis, duration of therapy, and cumulative intake of corticosteroids and other immunosuppressants. This study aims to analyze the auxological status of male and female JDMS patients from a tertiary care center in North-Western India and establish age and gender-specific database for physical growth parameters for JDMS children.

Our study cohort included children diagnosed with JDMS, with approximately 57% diagnosed between the ages of 3 and 7 years. Half of the patients had received treatment for more than six years. Notably, none of the parents of the subjects had a history or features of JDMS.

Growth patterns in JDMS patients

The growth patterns for both height and weight for JDMS boys and girls demonstrated a regular increase throughout the study period. The BMI growth patterns showed a continuous upward trend among female patients, while an undulating pattern was observed in the male cohort. Although statistically non-significant, the lower placement of growth curves for JDMS boys compared to their unaffected Indian counterparts [14] suggests that boys with JDMS experience a compromised auxological status.

In contrast, the height, weight, and BMI growth patterns for JDMS girls surpassed those of their un-affected Indian peers after 13 years, indicating that girls may be more resistant to the adverse effects of JDMS. Furthermore, female JDMS patients exhibited higher subcutaneous fat deposition, measured by skinfold thickness, than their male counterparts. Fat deposition was highest in the anterior thigh region and lowest at the biceps.

The better auxological performance of JDMS girls in this study, compared to boys, may be linked to the higher mean MMT-8 score observed in boys, which indicates greater muscle weakness. This was significantly correlated with height, weight, and BMI, while no such correlation was observed in females. However, non-significant gender differences in the anthropometric parameters could be attributed to the small sample size of the study.

Growth failure in JDMS

In our study, 6.6% of children showed growth failure, defined as a height below the 3rd centile of the IAP 2015 reference. This percentage is lower than the 21% and 15% growth failure rates reported in female and male JDMS patients, respectively, by Nordal et al. [8], and the 8% reported among a cohort of 424 JDMS patients across Latin America and Europe [7]. These authors opined that though there is marked improvement in the outcome of juvenile DM due to the advancement of medicine over the years yet, the chronic course was the strongest predictor of poor prognosis among these patients. The lower percentage of growth failure in our study may be attributed to demographic differences, as well as a smaller sample size. Our study also found that 31% of the children were considered short-statured according to their mid-parental height, though no studies currently assess the effect of JDMS on final adult height.

Obesity and metabolic implications

Approximately 40% of our study cohort were either obese (10%) or overweight (28.33%). This could be attributed to the prolonged use of corticosteroids, which are known to cause weight gain. These findings emphasize the need for monitoring steroid-induced morbidity and developing appropriate steroid-sparing strategies in JDMS patients. Unfortunately, we were unable to assess the effect of corticosteroid dosage on physical growth due to the unavailability of information.

It is clear from the above discussion that boys with JDMS showed compromised growth compared to their un-affected counterparts. This could be attributed to the influence of disease activity on the physical growth of male patients with JDMS. However, the better auxological prognosis observed for female JDMS patients beyond 12-13 years, when compared to their un-affected peers, suggests the existence of a protective mechanism favoring girls, making them more resistant to the adverse effects of the disease.

Strengths and limitations

A major strength of this study is the presentation of longitudinal growth velocities for various anthropometric variables in JDMS patients. However, the higher magnitude of variability encountered around the mean appears to be an artefact of small number of children afflicted with this disease in addition to their disease severity as well as its variable improvement patterns recorded during the study period.

Despite these strengths, there are limitations, including the small sample size and the skewed data. Due to unforeseen travel restrictions, clinic closures, reduction in staff, and other safety issues related to the COVID-19 pandemic during the study period, not all patients could be followed up as planned, leading to a substantial reduction in the sample size.

## Conclusions

The interplay of disease activity, chronic inflammation, and prolonged steroid therapy likely impacts growth in children with JDMS, especially boys. Nutritional and socioeconomic factors may also play a role. Therefore, regular growth monitoring and steroid-sparing strategies are essential for timely interventions to improve growth and pubertal status. The data provided highlights growth dynamics and creates a database for comparing JDMS patients across populations. Further longitudinal studies are needed to better understand the growth characteristics of these children.
